# plantsUPS: a database of plants' Ubiquitin Proteasome System

**DOI:** 10.1186/1471-2164-10-227

**Published:** 2009-05-16

**Authors:** Zhou Du, Xin Zhou, Li Li, Zhen Su

**Affiliations:** 1 State Key Laboratory of Plant Physiology and Biochemistry, College of Biological Sciences, China Agricultural University, Beijing, 100193, PR China

## Abstract

**Background:**

The ubiquitin 26S/proteasome system (UPS), a serial cascade process of protein ubiquitination and degradation, is the last step for most cellular proteins. There are many genes involved in this system, but are not identified in many species. The accumulating availability of genomic sequence data is generating more demands in data management and analysis. Genomics data of plants such as *Populus trichocarpa*, *Medicago truncatula*, *Glycine max *and others are now publicly accessible. It is time to integrate information on classes of genes for complex protein systems such as UPS.

**Results:**

We developed a database of higher plants' UPS, named 'plantsUPS'. Both automated search and manual curation were performed in identifying candidate genes. Extensive annotations referring to each gene were generated, including basic gene characterization, protein features, GO (gene ontology) assignment, microarray probe set annotation and expression data, as well as cross-links among different organisms. A chromosome distribution map, multi-sequence alignment, and phylogenetic trees for each species or gene family were also created. A user-friendly web interface and regular updates make plantsUPS valuable to researchers in related fields.

**Conclusion:**

The plantsUPS enables the exploration and comparative analysis of UPS in higher plants. It now archives > 8000 genes from seven plant species distributed in 11 UPS-involved gene families. The plantsUPS is freely available now to all users at .

## Background

The ubiquitin/26S proteasome system (UPS) is the major pathway of protein degradation. UPS can affect all aspects of cellular function, and plays an important role in physiological processes like hormonal responses, biotic stress and photomorphogenesis. In UPS, substrate proteins destined for degradation are tagged with 76-residue ubiquitin proteins through a serial cascade process of so-called ubiquitination, and finally hydrolysed by 26S proteasome. There are three steps in ubiquitination, catalyzed by three different enzymes or enzyme complexes: ubiquitin activating enzyme (E1), ubiquitin conjugating enzyme (E2), and ubiquitin protein ligase (E3). There are approximately 1300 E3s in the Arabidopsis genome, and similarly large numbers in other plants. However, in most plant species, the genome-wide classification and annotation of UPS genes, especially E3 families, are not yet available. The rapidly accumulating genome sequences has made those of seven important higher plants: Arabidopsis (*Arabidopsis thaliana*), rice (*Oryza sativa*), *Populus trichocarpa*, *Medicago truncatula*, grape (*Vitis vinifera*), soybean (*Glycine max*), and maize (*Zea mays*) publicly available. Consequently, analysis work is now inevitable and urgent. However, until now there was no available database concerning higher plants' UPS. The only comprehensive UPS database is PlantsUBQ [1], which provides information for only a single Arabidopsis species. To help researchers interested in plants' UPS, we developed the platform 'plantsUPS'. This archives > 8000 genes from the above seven plant species, belonging to 11 UPS gene families (one each for E1 and E2, and nine for E3).

## Construction and content

### Genome sequence data acquisition

Arabidopsis genome data used in plantsUPS is from TAIR ([2] release 8, and rice data is from the Rice Genome Annotation Project [3] release 5. *Populus*, soybean, grape, *Medicago *and maize genome data are compiled from the Populus genome project [4], Soybean Genome Project [5], Genoscope [6], MGSC [7] and MaizeSequence.org [8], respectively, and all used the latest versions available in February 2009. We used maize protein-coding genes for analysis; however, due to the highly complex and unfinished annotation of the maize genome, genes in plantsUPS should not be considered as an integrated UPS profile of maize.

### UPS genes identification

In plantsUPS, we used BLAST [9] and InterproScan [10] searches in computational prediction to identify UPS gene members for 11 gene families. We used BLAST (E-values ≤ 10.0) as the primary search before performing InterproScan. However, for RBX (Ring-Box) and DDB which is a component of CDD (CUL4-RBX1-CDD complex) families there is no consensus IPR (Interpro Scan) accession for identification. Thus only BLAST was used (E-value ≤ 1e–30). We used InterproScan results as the main evidence in estimation. Gene families and the corresponding IPR accessions used are presented in supplement Table S1 [see Additional file 1]. Subsequently, we did manual curation to reduce the false-positive rate, based on published reports.

### Database architecture

We constructed and configured plantsUPS upon a typical LAMP (Linux + Apache + MySQL + PHP) platform. Dataset was stored in MySQL 4.1 [see Additional file 2], and web interface was achieved by PHP scripts (PHP version 4.4) on Red Hat Linux, powered by an Apache server.

## Utility and discussion

### Web function and comparative tools

**Figure 1:**
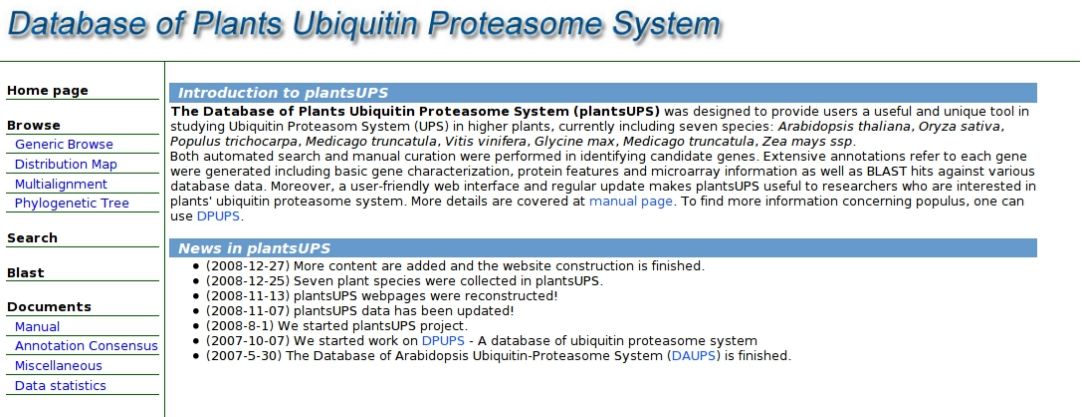
**plantsUPS index page**. The plantsUPS provides a user-friendly interface and various browsing or searching methods.

We designed a user-friendly website interface. Users can browse or search different levels of content taxa by various choices. Using the basic browse function, users can browse every species of UPS genes by setting a limit of gene family or chromosome number. Location distribution maps, multi-sequence alignments, and phylogenetic trees can also be browsed quickly (Figure 1). The chromosome maps containing gene loci located on the chromosomal linkages were generated and visualized using GenomePixelizer [11], which give users a direct scope to the distribution of UPS genes on chromosomes and are especially useful in observing tandem duplications [see Additional file 3]. The plantsUPS supports a comprehensive search function, for example, searching more than one species or gene family simultaneously, or specifying certain inquiries like gene model name, GO (gene ontology) ID or IPR accession. The association with a brief summary on the page can provide users a useful platform for searching and also for comparative study of different level information. For Arabidopsis and rice, we further provided a Gbrowser tool for comparative study of UPS and non-UPS genes, and provided a graphical way to explore expression of Arabidopsis genes under stress treatments.

### Extensive gene annotation

**Figure 2:**
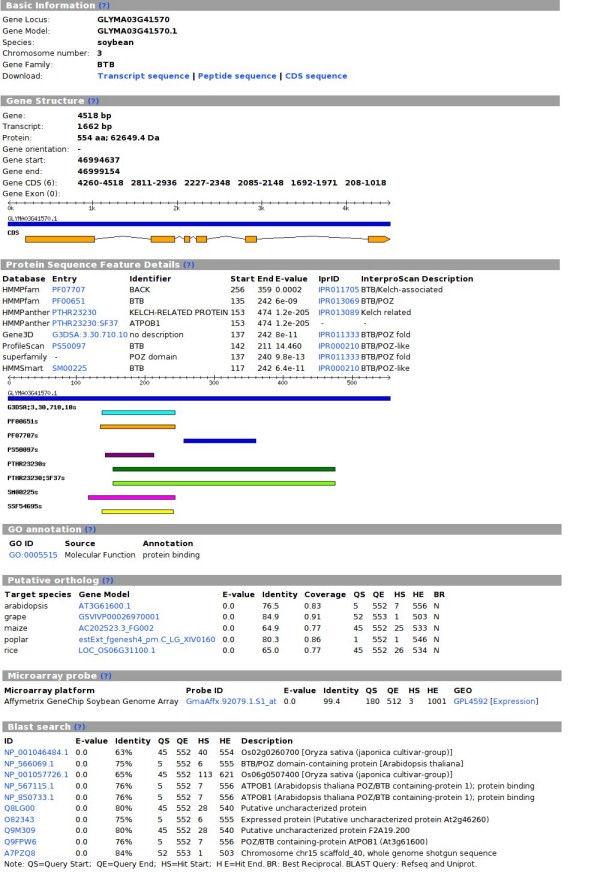
**Single gene browse**. Extensive annotations are presented in the single-gene display page. Here we use a gene from the soybean BTB family as an instance.

To better facilitate users inspecting each single UPS member, we collected and organized exhaustive information for genes in plantsUPS. In the gene browsing page (Figure 2), users can find a gene's basic information, gene and protein structure features with brief model figures, GO annotation, as well as identified probes on different microarray platforms. Also on this page, pre-computed top-matched hits by BLAST search against genes from plantsUPS, Refseq and UniProt databases can be useful for cross-organism study. The best reciprocal hits are marked for use in identifying putative orthologs.

### Microarray expression value

To explore expression of UPS genes, we retrieved and categorized microarray expression data from Gene Expression Omnibus (GEO) [12]. We picked out eight different GEO platforms (GPL) including 89 GEO series (GSE) data from 11 GPLs in plantsUPS (Table 1), and collected most parts of GSEs for these GPLs in GEO. For GPL198 of Arabidopsis, we mainly used AtGenExpress Consortium's data because of its high quality and experimental continuity. To provide better understanding, we classified 89 GSEs into five different aspects: tissue, stress, development, treatment and other, based on their experimental descriptions [see Additional file 4]. Users can directly inspect gene expression values by choosing any one of the above five classes.

**Table 1:** GPLs in plantsUPS

**Species**	**GPL**	**GSE number**
Arabidopsis	GPL198	28
Rice*	GPL2025, GPL892	24
Poplar	GPL4359	2
Maize	GPL4032	16
Grape	GPL1320	2
Soybean	GPL4592	12
Medicago	GPL4652	5

We collected eight different GEO platforms (GPLs) for seven species in plantsUPS including 89 GEO series.

*There are two GPLs from rice organism.

## Discussion

Six of the seven organisms contained > 1000 members (Table 2), except for grape. This result matched our primary expectation and infers the importance and complexity of UPS in plants. The UPS genes took up around 2.2% of genomic genes in most species, except Arabidopsis with much higher at 4.1%. At the gene-family level, F-box and RING finger families of E3 were the most abundant groups. In Xu's newly published paper [13], F-box genes in Arabidopsis, *Poplar *and rice were identified as 692, 337 and 779, respectively. These numbers are similar to our result in plantsUPS (654, 335 and 702, respectively), and the unmatched parts may be caused by applying different automated prediction methods or stringency criteria.

**Table 2:** Genes data statistics in plantsUPS

	**E1**	**E2**	**Fbox**	**SKP**	**RING finger**	**BTB**	**Cullin**	**Ubox**	**HECT**	RBX*	DDB*	**Total**
**Arabidopsis**	2	47	654	21	465	79	10	62	7	2	5	1354
**Rice**	6	49	702	27	378	126	10	76	8	2	3	1387
**Poplar**	6	70	335	15	399	81	13	93	7	3	5	1027
**Soybean**	6	107	418	18	725	77	24	120	19	2	3	1519
**Grape**	3	45	153	10	330	63	8	56	9	null	null	677
**Medicago**	2	40	539	36	294	41	12	38	9	null	null	1011
Maize**	9	59	325	31	401	129	6	119	21	null	null	1100

Six species have > 1000 UPS members. F-box and RING finger families are the most abundant gene families.

*BLAST search for RBX or DDB families may find no qualified hits in some species, and we present 'null' for such situations.

**For reasons of ongoing annotating and unexpected complexity in maize, the numbers here may not reflect the integrated UPS profile for maize.

**Figure 3:**
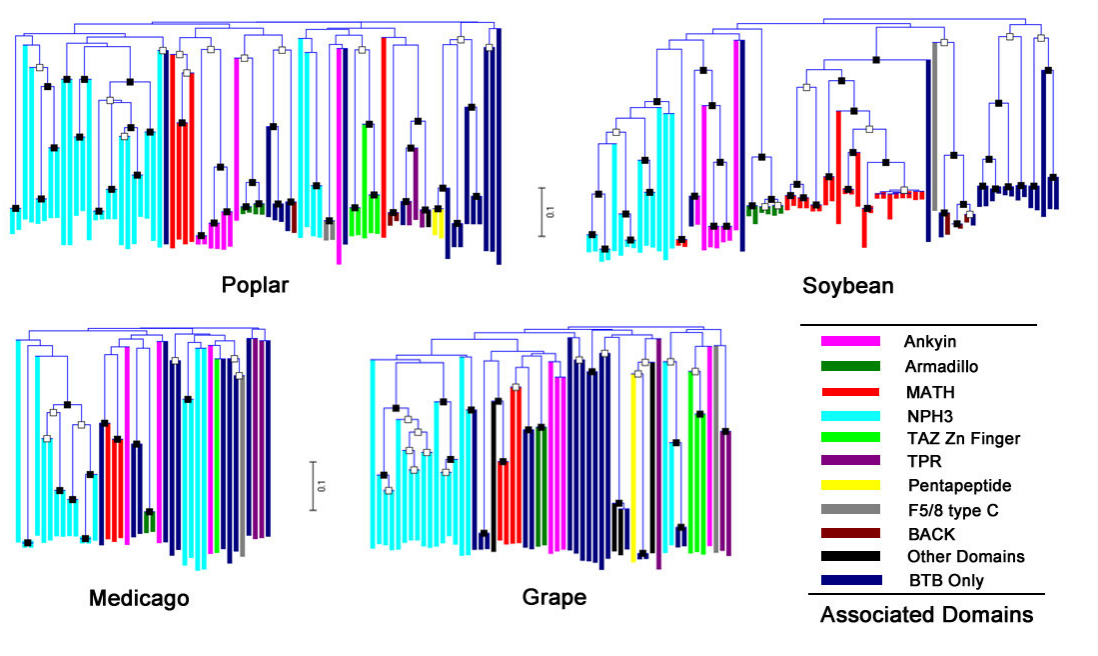
**Phylogenetic trees of *Poplar*, grape, *Medicago *and soybean BTB protein families**. BTB domains in the BTB family of four organisms identified by InterproScan were used to generate a midpoint-rooted NJ (neighbor joining) tree. The trees were created by Mega 4.0 with 1000 bootstrap replicates. Solid boxes on the nodes of the trees present higher bootstrap support (≥ 90%), and hollow ones indicate moderate (≥ 60%). Individual branches are color-coded by the nature of protein domains. We characterized all BTB proteins into 11 classes. Expanded views of trees with more detail can be found in supplement Figures S3–6 [see Additional file 5, 6, 7, 8].

We chose the BTB (Broad-complex, Tramtrack, Bric-a-Brac) family from *Poplar*, *Medicago*, grape and soybean for phylogenetic tree analysis (Figure 3) [also see Additional file 5, 6, 7, 8]. Individual members of the trees were further clustered and color-coded, based on the nature of protein domains/motifs. There were 11 classes characterized: ankyin, armadillo, meprin and TRAF homology (MATH), NPH3, TAZ type Zn Finger, tetratricopeptide (TRP) repeats, pentapeptide, F5/8 type C domain, BTB/Kelch-associated (BACK) domain, other domains, and BTB domains only. Ankyin, armadillo, MATH, NPH3 and F5/8 type C domains were recognized in all four organisms appended to the BTB domain, while other domains may be absent in one or two species. The numbers of MATH domains in *Poplar *and *Medicago *were similar to previous results [14], but interestingly as many as 24 MATH-domain-containing genes were identified in the dicotyledon soybean.

In the future, we will continue to incorporate new information, develop more comparative tools for plantsUPS, and extend the available species. Regular update and relative analysis will provide users up-to-date UPS information.

## Conclusion

The plantsUPS is the first platform concerning UPS in seven sequenced higher plants. It will assist searchers in related fields by providing comprehensive information on UPS gene families and members of these families. The plantsUPS resource is freely available via .

## Authors' contributions

ZD performed data collection and annotation, the database and web server construction, and compiled the first draft of the manuscript. XZ provided system support and LL constructed the Arabidopsis UPS web server template. ZS supervised the project. All authors read and approved the final manuscript.

## Supplementary Material

Additional file 1 **Supplement Table S1. IPR accessions for each gene family used in identifying BLAST and InterproScan search are used in identifying genes involved in UPS**. The IPR accessions presented were mainly used for different families. No consensus IPR accession was plausible for RBX and DDB families, thus we mainly used BLAST search in these cases. Click here for file

Additional file 2 **Supplement Figure S1. MySQL database structure model for plantsUPS**. We use MySQL 4.1 to store our dataset. Click here for file

Additional file 3 **Supplement Figure S2. Distribution map of soybean**. The distribution map presents the locations of soybean UPS genes. Genes are represented by squares and color coded according to their gene families. Clicking any block will redirect to the corresponding gene or gene-family browsing web page. Click here for file

Additional file 4 **Supplement Table S2. The 89 GSEs collected in plantsUPS**. We complied 89 GSEs from GEO, and manually categorized them into five different classes. Click here for file

Additional file 5 **Supplement Figure S3. Phylogenetic tree of soybean BTB protein family**. Expanded views of phylogenetic tree with sequence identifiers in non-topology type for soybean BTB proteins. Click here for file

Additional file 6 **Supplement Figure S4. Phylogenetic tree of *Poplar *BTB protein family**. Expanded views of phylogenetic tree with sequence identifiers in non-topology type for *Poplar *BTB proteins. Click here for file

Additional file 7 **Supplement Figure S5. Phylogenetic tree of *Medicago *BTB protein family**. Expanded views of phylogenetic tree with sequence identifiers in non-topology type for *Medicago *BTB proteins. Click here for file

Additional file 8 **Supplement Figure S6. Phylogenetic tree of grape BTB protein family**. Expanded views of phylogenetic tree with sequence identifiers in non-topology type for grape BTB proteins. Click here for file
