# Supplementary material for: plantsUPS: a database of plants' Ubiquitin Proteasome System
Source: BMC Genomics. 2009 May 16;10:227. doi: 10.1186/1471-2164-10-227 (PMC2690602; doi:10.1186/1471-2164-10-227)
Supplement: Additional file 1 — Supplement Table S1. IPR accessions for each gene family used in identifying BLAST and InterproScan search are used in identifying genes involved in UPS. The IPR accessions presented were mainly used for different families. No consensus IPR accession was plausible for RBX and DDB families, thus we mainly used BLAST search in these cases. [file 1471-2164-10-227-S1.doc]

**Additional table 1**- IPR accessions for each gene family used in identifying BLAST and InterproScan search are used in identifying genes involved in UPS. The table presents IPR accessions we mainly used for different families, while since no consensus IPR accession is plausible for RBX and DDB family we mainly use BLAST search for this two families.

| **Gene family in UPS** | **IPR accession number** |
| --- | --- |
| E1 | IPR000011 |
| E2 | IPR000608 |
| Fbox | IPR001810 |
| RING finger | IPR001841 |
| HECT | IPR000569 |
| BTB* | IPR013069 |
| Ubox | IPR003613 |
| Cullin | IPR001373 |
