# Supplementary figures and images for: plantsUPS: a database of plants' Ubiquitin Proteasome System
Source: BMC Genomics. 2009 May 16;10:227. doi: 10.1186/1471-2164-10-227 (PMC2690602; doi:10.1186/1471-2164-10-227)

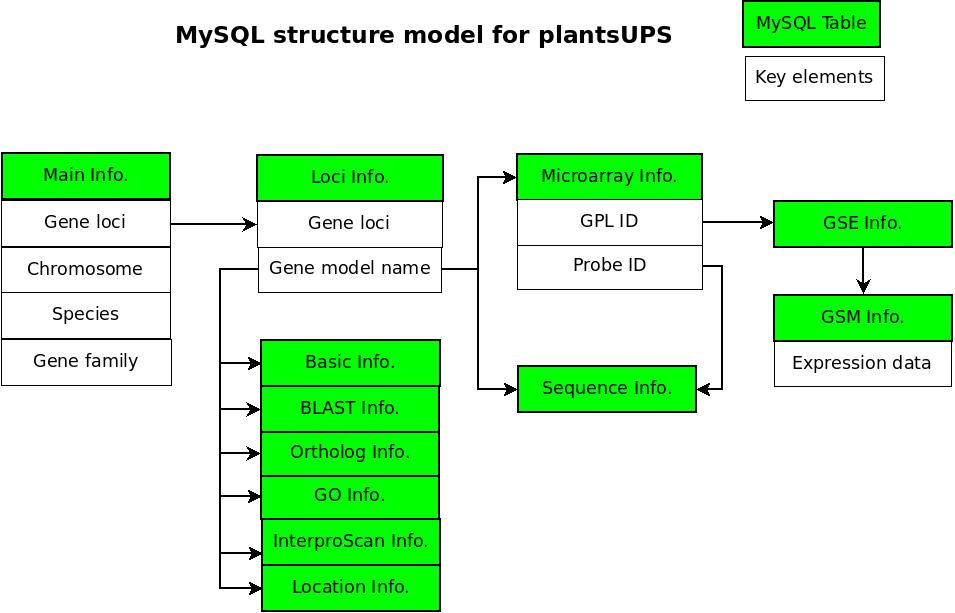

Supplement: Additional file 2 — Supplement Figure S1. MySQL database structure model for plantsUPS. We use MySQL 4.1 to store our dataset. [file 1471-2164-10-227-S2.jpeg]

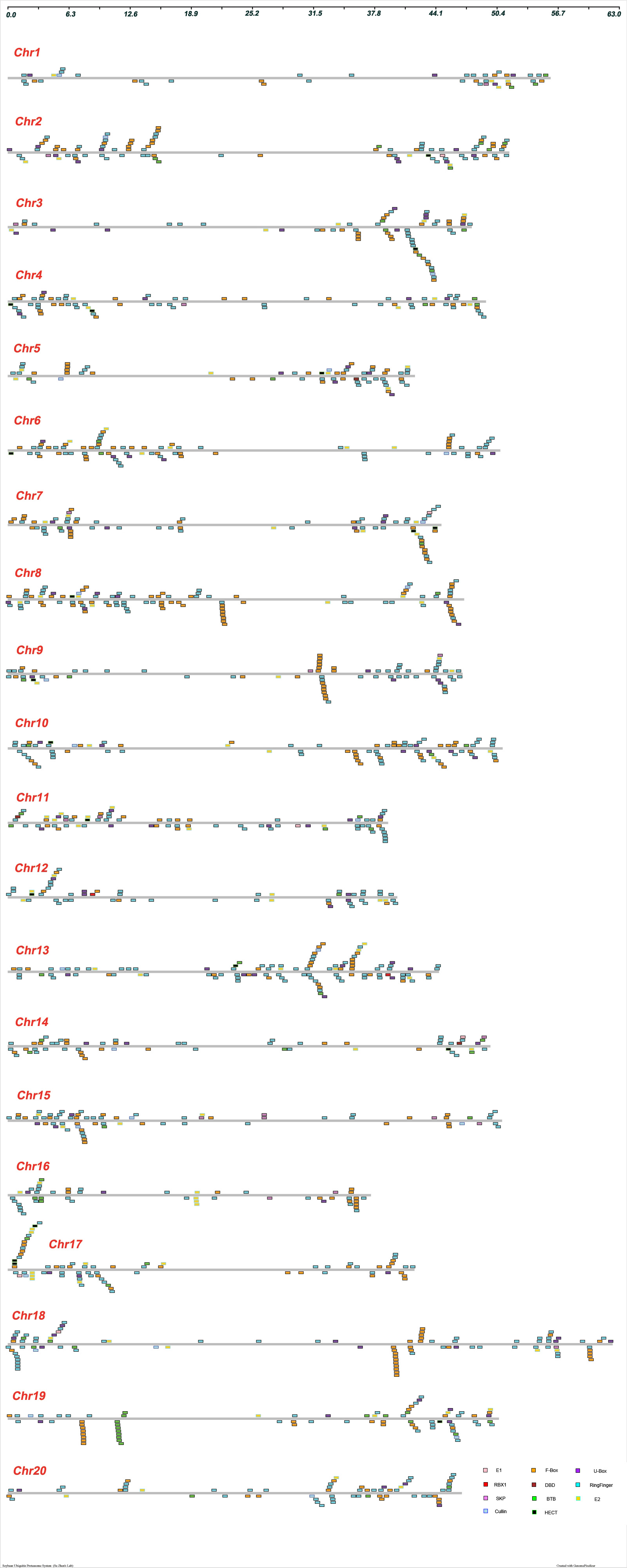

Supplement: Additional file 3 — Supplement Figure S2. Distribution map of soybean. The distribution map presents the locations of soybean UPS genes. Genes are represented by squares and color coded according to their gene families. Clicking any block will redirect to the corresponding gene or gene-family browsing web page. [file 1471-2164-10-227-S3.jpeg]

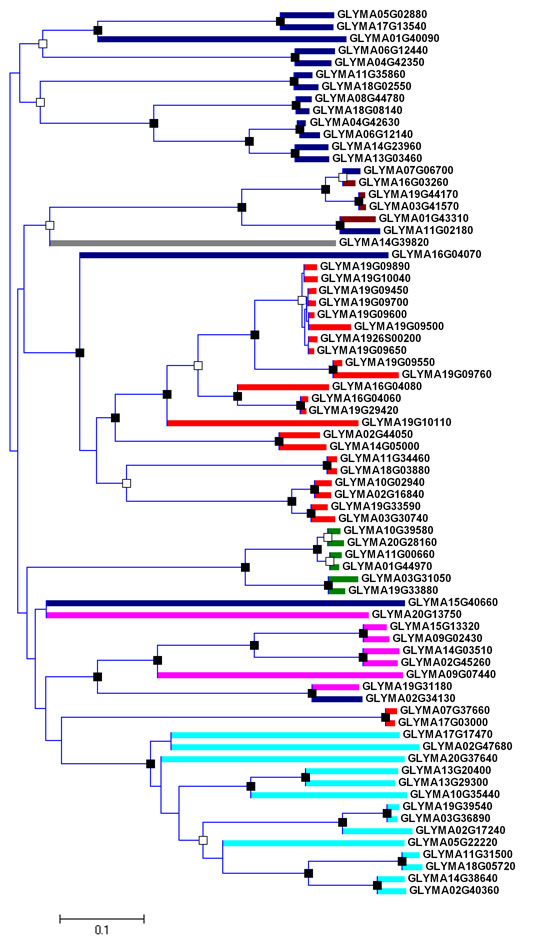

Supplement: Additional file 5 — Supplement Figure S3. Phylogenetic tree of soybean BTB protein family. Expanded views of phylogenetic tree with sequence identifiers in non-topology type for soybean BTB proteins. [file 1471-2164-10-227-S5.jpeg]

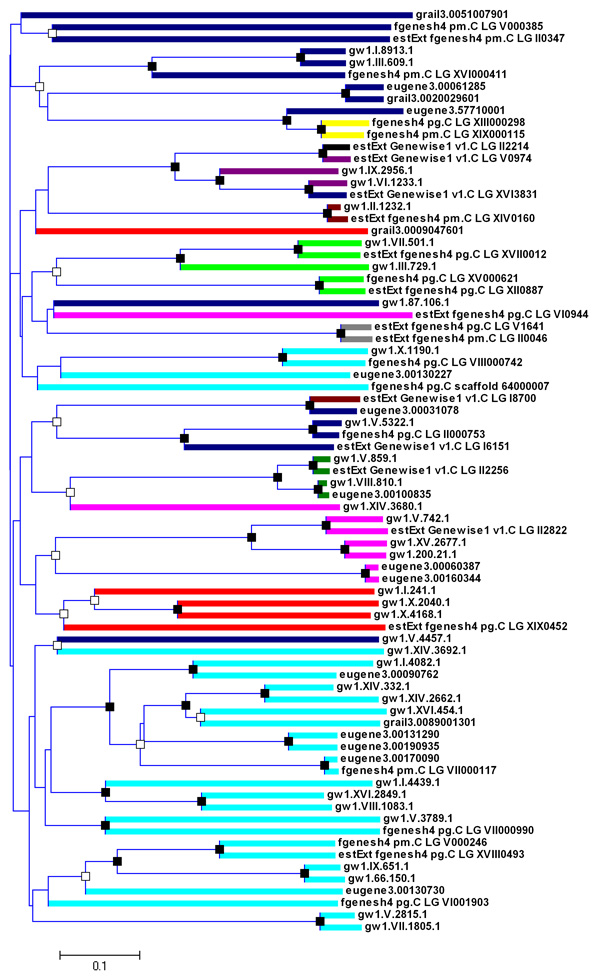

Supplement: Additional file 6 — Supplement Figure S4. Phylogenetic tree of Poplar BTB protein family. Expanded views of phylogenetic tree with sequence identifiers in non-topology type for Poplar BTB proteins. [file 1471-2164-10-227-S6.jpeg]

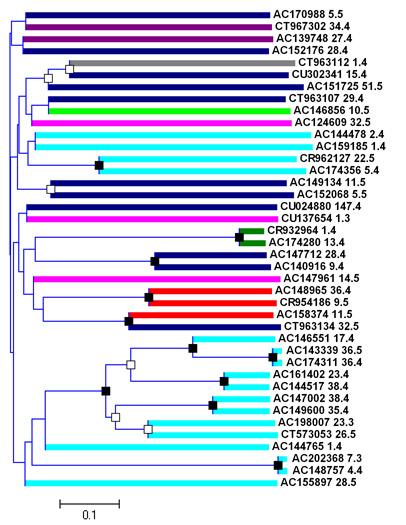

Supplement: Additional file 7 — Supplement Figure S5. Phylogenetic tree of Medicago BTB protein family. Expanded views of phylogenetic tree with sequence identifiers in non-topology type for Medicago BTB proteins. [file 1471-2164-10-227-S7.jpeg]

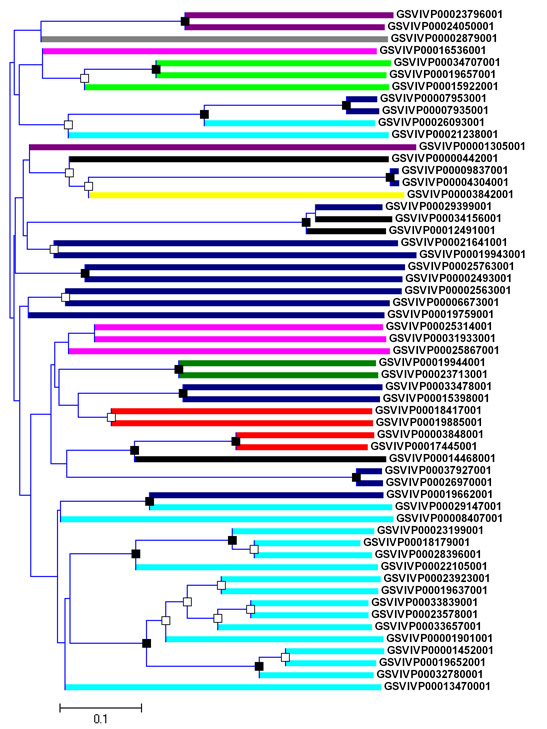

Supplement: Additional file 8 — Supplement Figure S6. Phylogenetic tree of grape BTB protein family. Expanded views of phylogenetic tree with sequence identifiers in non-topology type for grape BTB proteins. [file 1471-2164-10-227-S8.jpeg]
